# Vegetables, fruits, and berries – a scoping review for Nordic Nutrition Recommendations 2023

**DOI:** 10.29219/fnr.v68.10455

**Published:** 2024-01-25

**Authors:** Magdalena Rosell, Lars T. Fadnes

**Affiliations:** 1 Department of Biosciences and Nutrition, Karolinska Institutet, Stockholm, Sweden; 2 Department of Global Public Health and Primary Care, University of Bergen, Bergen, Norway; 3 Bergen Addiction Research, Department of Addiction Medicine, Haukeland University Hospital, Bergen, Norway

**Keywords:** vegetables, fruits, berries, dietary guidelines

## Abstract

Vegetables, fruits, and berries comprise a large variety of foods and are recognised to play an important role in preventing chronic diseases. Many observational studies have been published during the last decade, and the aim of this scoping review is to describe the overall evidence for the role of vegetables, fruits, and berries for health-related outcomes as a basis for setting and updating food-based dietary guidelines. A scoping review was conducted according to the protocol developed within the Nordic Nutrition Recommendations 2023 project. Current available evidence strengthens the role of consuming vegetables, fruits, and berries in preventing chronic diseases. The most robust evidence is found for cancer in the gastric system and lung cancer, cardiovascular disease, and all-cause mortality. Steeper risk reductions are generally seen at the lower intake ranges, but further reductions have been seen for higher intakes for cardiovascular disease. Weaker associations are seen for type 2 diabetes. There is evidence that suggests a beneficial role also for outcomes such as osteoporosis, depression, cognitive disorders, and frailty in the elderly. The observed associations are supported by several mechanisms, indicting causal effects. Some subgroups of vegetables, fruits, and berries may have greater benefits than other subgroups, supporting a recommendation to consume a variety of these foods.

## Popular scientific summary

- Vegetables, fruits, and berries are commonly high in water, low in energy, and are good sources of fibre, vitamins, minerals and other bioactive compounds.
- The average intake of vegetables, fruits, and berries in the Nordic and Baltic countries is generally below 400 g per day.
- Recent high-quality evidence supports the role of consuming vegetables, fruits, and berries in the prevention of chronic diseases.
- The evidence is strongest for gastric and lung cancer, cardiovascular disease, and all-cause mortality.
- Consuming a variety of vegetables, fruits, and berries should be recommended.

Vegetables, fruits, and berries comprise a large variety of foods. They have more exact botanical definitions; however, culinary definitions are more commonly used in nutrition research since they better correspond with how people think of these foods (1). Vegetables are accordingly defined as edible parts of plants, such as leaves, roots, tubers, bulbs, stems and stalks and flowers. They also include some foods botanically defined as grains, such as sweetcorn, or as fruits, such as cucumber, pepper, tomato, aubergine, and courgette. Fruits can be culinarily defined as the edible flesh that surrounds the seeds produced by a tree or other plant that has a sweet or tart taste (1), while berries can be defined as small, fleshy fruits, often juicy and brightly coloured (2). Legumes, potatoes, and fruit juice are food groups that will not be covered in the present review.

Although vegetables, fruits, and berries comprise a heterogeneous food group from a nutritional point of view, they are commonly high in water, low in energy, contain numerous nutrients, and are good sources of fibre, vitamin C, vitamin E, vitamin K, folate, and potassium. They also contain a vast range of other bioactive compounds, such as phytochemicals, and the synergistic effects of these are still not fully understood (3, 4).

Vegetables can be further divided into different subgroups based on botanic family, colour, part of plant or content of nutrients and other bioactive compounds (5, 6). A commonly used subgroup is cruciferous vegetables (Brassica), such as broccoli, brussels sprouts, cabbage, cauliflower, kale, and turnips, which are also sources of calcium and selenium and have gained increased attention due to their high content of organosulfur compounds and their possible health effects (6). Leafy green vegetables such as spinach, Swiss chard, and lettuce, comprise another subgroup characterised by their content of nitrate, vitamin K, iron, zinc, calcium and magnesium as well as carotenoids and flavonoids (6), with particularly high concentrations of carotenoids found in dark green leafy vegetables (6). Yellow-orange-red vegetables, such as tomato, carrot, pumpkin, and yellow and red pepper, comprise yet another subgroup rich in carotenoids, while allium vegetables, such as onion, garlic and leek are characterised by a high content of organosulfur compounds and flavonoids (6). Due to the higher content of starch, some tubers and roots, such as potatoes, sweet potatoes and cassava, are classified as starchy vegetables, separated from non-starchy root vegetables, such as carrots, beets, parsnips, turnips, and swedes (1). Starchy vegetables are an important source of energy in the diet and are usually not included in the classification of vegetables (1). Legumes are also often considered a separate food group.

Subgroups of fruits include, for example the citrus family, such as orange, lemon, grapefruit, and tangerine, which are particularly rich in vitamin C and also contain flavonoids, alkaloids, coumarins, phenolic acids, carotenoids, and limonoids (7). Pome fruits, such as apples and pears, have also been associated with health benefits, possibly due to their content of flavonoids and other antioxidants (8). Berries, such as blueberries, strawberries, blackberries, and cranberries, stand out with a particularly high content of phytochemicals such as flavonoids, including anthocyanins, ellagitannins, and phenolic acids (9, 10).

The classifications of vegetables, fruits and berries may vary in different countries. For example, legumes have been included in the vegetable group in former dietary guidelines in Finland and Denmark, and fruit juice have been included in the fruit group in Norway and Denmark, while this has not been the case in Sweden. Potatoes, legumes, and fruit juice are not discussed in this review. However, they may have been included in individual studies investigating health effects of vegetables since the definitions may differ and are not always clarified.

Vegetables, fruits, and berries have been recognised to play an important role in preventing chronic diseases, and dietary guidelines generally encourage a high intake of these foods (11). The body of evidence is growing, and many cohort studies examining long-term effects have been published during the last decade. The aim of this scoping review is to describe the overall evidence for the role of vegetables, fruits, and berries for important health-related outcomes, including chronic diseases with substantial morbidity, as a basis for setting and updating food-based dietary guidelines (FBDGs) (Box 1). The search strategy was focused on systematic reviews and meta-analyses of observational and intervention studies summarising epidemiologic evidence in this area.

*Box 1.* Background papers for Nordic Nutrition Recommendations 2023

- This paper is one of many scoping reviews commissioned as part of the Nordic Nutrition Recommendations 2023 (NNR2023) project (12).
- The papers are included in the extended NNR2023 report but, for transparency, these scoping reviews are also published in Food & Nutrition Research.
- The scoping reviews have been peer-reviewed by independent experts in the research field according to the standard procedures of the journal.
- The scoping reviews have also been subjected to public consultations (see report to be published by the NNR2023 project).
- The NNR2023 committee has served as the editorial board.
- While these papers are a main fundament, the NNR2023 committee has the sole responsibility for setting dietary reference values in the NNR2023 project.

## Methods

This scoping review follows the protocol developed within the NNR2023 project (12). Sources of evidence used in the review follow the eligibility criteria described previously (13). One qualified systematic review (qSR) was identified for this review, namely the Continuous Update Project Expert Report 2018 from the World Cancer Research Fund/American Institute of Cancer Research (WCRF/AICR) on wholegrains, vegetables and fruit, and the risk of cancer (1). No *de novo* NNR2023 systematic review was conducted on this topic (14). To summarise the evidence regarding the association between the intake of vegetables, fruits, and berries and outcomes other than cancer, a literature search for systematic reviews and meta-analyses was performed in PubMed/MEDLINE using the following search string:

(fruit*[Title/Abstract] OR vegetable*[Title/Abstract] OR berry[Title/Abstract] OR berries[Title/Abstract] OR potato*[Title/Abstract] OR “Fruit”[Mesh Terms:noexp] OR “Vegetables”[Mesh Terms:noexp] OR “Fruit and Vegetable Juices”[Mesh]) AND (2011:2023[pdat]) AND (meta-analysis[Filter] OR systematicreview[Filter])

The main search was conducted on the 4th of April 2021, and an updated search was conducted on the 28th of February 2023. Inclusion and exclusion criteria were used based on relevance and importance in relation to FBDGs. Systematic reviews of systematic reviews were also included and checked regarding their references. This resulted in a total of 1,671 references, limited to 303 based on titles, to 207 based on abstracts, and to 166 based on assessment of full articles (supplementary file). Reference lists were also checked, and two additional articles were added based on cross-references. The articles were categorised according to outcomes, study designs, age groups, and subgroups of vegetables, fruits, and berries, when applicable. For the summary below, articles were selected from each category based on most recent reviews, comprehensiveness, or studies on new associations, and quality checked by using the modified AMSTAR 2-NNR tool (12, 15). Systematic reviews and meta-analyses of prospective studies and randomised controlled trials were of primary interest, but cross-sectional studies were also considered if no other evidence was available. For the section on mechanisms, a general search approach was also used to complement the systematic reviews and meta-analyses on intermediate risk markers.

## Dietary intake in Nordic and Baltic countries

According to data from national dietary surveys in adults, the mean intake of vegetables, fruits, and berries ranges from around 200 to 400 g per day among the different Nordic and Baltic countries, with the lowest intakes seen in Iceland and the highest in Denmark (16). The mean intake of vegetables (potatoes not included) is generally between around 150 and 200 grams per day, with the lowest intakes seen in men and women in Iceland and the highest in women in Denmark and men in Latvia. The mean intake of fruits and berries is generally between 100 and 200 g per day, with the lowest intakes seen in men in Iceland and Sweden and the highest in women in Denmark and Estonia. In all eight countries, the intakes of fruit and berries are higher in women than in men, while the differences between the sexes are generally smaller and inconsistent regarding the intake of vegetables (16).

## Health outcomes relevant for Nordic and Baltic countries

Most meta-analyses and systematic reviews have not investigated berries separately from fruits, and the results below are therefore reported for fruits in general unless otherwise stated. It is also generally not stated whether fruits and vegetables include legumes, potatoes, and fruit juice. However, according to meta-analyses that have looked at subgroups of fruits and vegetables, legumes are generally not included while fruit juice and sometimes potatoes may be included.

The Continuous Update Project Expert Report from WCRF/AICR (2018), representing the only qSR eligible for this scoping review, concludes that intake of non-starchy vegetables and fruit probably decreases the risk of most cancers in the gastric system and lung (1). When non-starchy vegetables and fruit are investigated as separate food groups in relation to specific types of cancers, the evidence is limited-suggestive. Subgroups of fruit and vegetables that may be protective for some types of cancers include citrus fruit and foods containing carotenoids, β-carotene, vitamin C, and isoflavones. Since the pattern of association is consistent, the aggregated evidence of a general protective effect of non-starchy vegetables and fruit is judged as probable, that is, strong (1).

Regarding evidence for other outcomes than cancer, recent meta-analyses have shown inverse associations between total fruit and vegetable intake combined as well as separately and cardiovascular disease incidence and mortality, including coronary heart disease and stroke (17–20). The risk reductions for high versus low intakes are generally in the range of 10–20%. The risk reductions are steeper at the lower intake ranges; however, the lowest risks were still seen in the highest ranges in some analyses (18, 19), corresponding to intakes of 800 g of fruit and vegetables per day (19). Analyses of subgroups of fruit and vegetables indicate that green leafy vegetables, cruciferous vegetables, allium, pome fruit (apples/pears), and citrus fruit may play a role in the observed risk reductions (18, 19). No associations were seen for berries. However, more evidence is needed to establish the role of specific fruits and vegetables. Inverse associations are also seen for all-cause mortality (19, 21, 22), with the maximum risk reductions plateauing at around 5–6 servings of fruit and vegetables per day, or around 2–3 servings of fruits per day and around 3–4 servings of vegetables per day (corresponding to around 400–480 g, 160–240 g, and 240–320 g, respectively, based on standard portion size of 80 g) (22), with no apparent increased risk reduction at intakes above this in the most recent meta-analysis (22). Analyses of subgroups of fruit and vegetables indicated inverse associations for green leafy vegetables, cruciferous vegetables, apples/pears, citrus fruit, and also berries (19). Again, these subgroup analyses are based on few studies.

For type 2 diabetes, the most recent and comprehensive meta-analysis reported a weak inverse association for the intake of fruit and vegetables separately as well as combined (23). Comparing high and low intakes, risk reductions of 7% were seen for fruit and for fruit and vegetables combined, and a non-significant trend of 5% risk reduction for vegetables. Non-linear dose-response analysis showed a risk reduction of around 10% from an intake of fruit and vegetables of 600–700 g/day compared with 0 g/day, although this was marginally statistically significant (23). Inverse associations were indicated for some fruits and berries, such as apples, blueberries, grapes, and raisins. Positive associations were observed for some foods, such as cantaloupe, and some types of cruciferous vegetables, however, not for boiled potatoes or cruciferous vegetable intake overall. However, the number of studies on subgroups of fruits and vegetables are small and some findings may have been due to selective reporting or chance. Using the WCRF criteria, a causal relationship was considered to be probable for fruit, limited-suggestive for fruit and vegetables combined, and limited – no conclusion for vegetables (23).

Regarding bone health, inverse associations have been reported between the intake of fruit and vegetables and the risk of hip fractures (24). This meta-analysis also included dietary patterns rich in fruit and vegetables, although adjustments were made for total nutrient intake, and the quality of evidence using the GRADE criteria was considered moderate (24). Another meta-analysis on case-control and cross-sectional studies demonstrated an inverse association between the intake of fruit only and postmenopausal osteoporosis (25). For vegetables, a similar association was seen in case-control studies, but not in cross-sectional studies (25).

Systematic reviews and meta-analyses are also found for other outcomes. Reduced risks of depression have been demonstrated (26–28), with risk reductions of around 10–15% comparing high and low intakes of fruit and vegetables separately (27, 28). A lower risk of cognitive disorders such as Alzheimer’s disease, dementia, and cognitive impairment has also been reported (29), although this meta-analysis was limited by considerable heterogeneity, which might be attributed to ethnic differences, and possible publication bias (29). The intake of fruits and vegetables was inversely associated with the risk of frailty (30) and positively associated with better muscle strength and function (31) in middle-aged and/or older adults. Inverse associations between the intake of fruits and vegetables and kidney stones are also indicated (32). The overall rating of systematic reviews and meta-analyses of prospective observational studies on the main outcomes mentioned above, using the modified AMSTAR 2-NNR, is presented in Table 1.

**Table 1:** The overall rating of systematic reviews and meta-analyses of prospective studies on the main outcomes mentioned above, using the modified AMSTAR 2-NNR (12)

Author, year	Number of prospective studies	Exposure	Outcome	Overall rating of the systematic review/meta-analysis
Bechthold et al. 2019 (17)	*n* = 19 (vegetables) *n* = 17 (fruits)	Fruits and vegetables	Cardiovascular disease	High confidence
Zurbau et al. 2020 (18)	*n* = 81	Fruits and vegetables	Cardiovascular disease	Moderate confidence
Aune, et al. 2017 (19)	*n* = 95	Fruits and vegetables	Cardiovascular disease All-cause mortality	High confidence
Schwingshackl et al 2017 (21)	*n* = 37 (vegetables) *n* = 34 (fruits)	Fruits and vegetables	All-cause mortality	High confidence
Wang et al. 2021 (22)	*n* = 24	Fruits and vegetables	All-cause mortality	Moderate confidence
Halvorsen et al. 2021 (23)	*n* = 23	Fruits and vegetables	Type 2 diabetes	High confidence
Brondani et al. 2019 (24)	*n* = 6	Fruits and vegetables	Bone fractures	High confidence
Dharmayani et al. 2021 (26)	*n* = 12	Fruits and vegetables	Depressive symptoms	High confidence
Matison et al. 2021 (27)	*n* = 4	Fruits and vegetables	Depression	High confidence
Ghoreishy et al. 2021 (30)	*n* = 10	Fruits and vegetables	Frailty	High confidence

Reduced risks of chronic obstructive pulmonary disease (33) and gallstone disease (34) have also been reported; however, the latter also included case-control studies and cross-sectional studies (34). Inverse associations between the intake of fruits and vegetables and the risk of inflammatory bowel disease (Crohn’s disease and ulcerative colitis) are also indicated (35, 36), of which one report was based on case-control studies only (36). A reduced risk has also been seen for periodontal diseases, but this was based on only two cohort studies on the elderly in Japan (37). Reduced risks are suggested for age-related cataract (only vegetable intake was investigated) (38) and macular degeneration (39), although the latter was of borderline statistical significance. Inverse associations between the intake of fruit and vegetables and risk of asthma and wheezing have also been reported in adults and children, primarily based on cross-sectional studies (40).

Looking at risk factors for diseases, meta-analyses of observational studies have suggested a positive association between the intake of fruit and vegetables and a reduced risk of overweight/obesity and weight gain (41, 42); however, the quality of evidence was considered low to moderate. The intake of fruit (43, 44) and/or vegetables (45) has also been associated with a reduced risk of hypertension, although the quality of evidence was considered low or very low (43). Inverse associations between the intake of fruit and/or vegetables and the metabolic syndrome have also been reported (46–48); however, two of these reports are mainly based on cross-sectional studies (47, 48).

Systematic reviews and meta-analyses of trials investigating specific vegetables, fruits or berries in concentrated forms, such as juice, purée, or powder, have shown beneficial effects on inflammatory biomarkers (49, 50), cardiovascular risk factors (50–54), and antioxidant status (55), although the evidence is not conclusive. These trials involve different study populations and may involve subjects with existing risk factors. The intake of berries or berry-based products may also have beneficial effects on cognitive function (56, 57).

Few trials have investigated vegetables, fruits, and berries as a food group. Some meta-analyses have shown that promoting or providing fruits and vegetables without any other interventions have no or possibly a slightly beneficial effect on body weight (58, 59), and may have beneficial effects on cardiovascular risk factors (60). However, these meta-analyses are based on very few studies and updated analyses are warranted.

## Mechanisms

The associations indicating long-term health benefits from vegetables, fruits, and berries seen in observational studies and trials are supported by several mechanisms related to different properties of these foods. The high content of water and low content of energy may lead to a decreased total energy intake and thereby contribute to control of body weight, although this effect could also partly be due to a replacement of less healthy foods in the diet (61). Vegetables, fruits, and berries also contain insoluble and soluble dietary fibre, which has documented health effects via a range of mechanisms (62, 63). Insoluble fibre reduces transit time and increases stool bulk, thereby preventing constipation (63). Soluble fibre forms gels when consumed, leading to a slower gastric emptying, maintained level of satiety, and attenuated postprandial changes in blood glucose and lipoprotein levels (63). In the large intestine, these fibres are also fermented to short-chain fatty acids, which decrease cholesterol synthesis and increase the excretion of bile (63), thereby reducing circulating blood cholesterol, and may also be protective against systemic inflammation (64). That soluble fibres have beneficial effects on lipid profiles and inflammatory markers is also supported by long-term randomised trials (65). Dietary fibre also affects the gut microbiota, acting as a prebiotic promoting the growth of favourable bacteria with positive effects on host nutrition and immunity (63).

The health benefits from vegetables, fruits and berries have also been suggested to be attributable to the content of antioxidant nutrients such as vitamin E, vitamin C, β-carotene, selenium, zinc, and other nutrients such as folate in these foods (61). However, randomised trials investigating possible effects of supplements of these nutrients have so far generally failed to show protective effects on cardiovascular disease, diabetes, or cancer (61, 66), indicating that the health effects of these foods cannot be explained by single nutrients, but probably by the combined effects of a range of constituents. Apart from the established nutrients, plant foods contain thousands of other bioactive compounds, or phytochemicals, including phenolics (such as flavonoids), alkaloids, organosulfur compounds, nitrogen-containing compounds, phytosterols, and carotenoids (6). More than 20,000 phytochemicals have been identified and a large proportion is still unknown (6). Many of these compounds possess strong antioxidant and free radical scavenging abilities and may therefore play an important role in many chronic diseases (3). Anti-inflammatory, anti-carcinogenic, anti-obesogenic, and anti-microbial effects have also been demonstrated both *in vitro* and *in vivo* (67–70). The suggested mechanisms are numerous and include effects on enzyme activities, signalling pathways and gene expressions. These compounds also interact with each other and the knowledge of how their bioavailability and bioactivity are affected when consuming mixtures of these and the effect of different food matrices is limited (4).

Vitamin C as well as total carotenoids and different types of carotenoids in blood serum may also be used biomarkers for fruit and vegetable intake (71). A comprehensive meta-analysis showed inverse associations between these biomarkers and the risk of cardiovascular disease, cancer, and all-cause mortality (72), and a large European cohort study (the EPIC study) showed an inverse association between these biomarkers and incident type 2 diabetes (73). Favourable associations between biomarkers of fruit and vegetable intake and gut bacterial composition and diversity have also been reported (74).

## Food-based dietary guidelines

NNR2012 concluded that increasing the intake of vegetables, fruits, and berries would potentially promote energy balance and health in the Nordic populations, primarily based on evidence regarding cardiovascular disease and cancer in the gastric system and lung. Current available evidence published since then strengthens the role of consuming vegetables, fruits, and berries for preventing chronic diseases. Most robust evidence is found for cancer in the gastric system and lung cancer, based on a qSR, as well as cardiovascular disease and all-cause mortality. Weaker effects are seen for type 2 diabetes. There are also studies that support a beneficial role of fruit and vegetable consumption for other outcomes such as osteoporosis, depression, cognitive disorders, and frailty in the elderly, which are also outcomes of public health concern in the Nordic and Baltic countries. There are also indications of beneficial effects on additional outcomes, although the evidence of these is generally limited.

The systematic reviews and meta-analyses on observational studies presented in the summary are predominantly based on prospective cohort studies unless otherwise stated, which excludes the possibility of a reverse direction of causality. The assessment of dietary intake is also not affected by the outcome, which may be the case in case-control and cross-sectional studies. However, confounding factors are always concerning since these may provide other explanations for the observed associations. Possible factors associated with fruit and vegetable intake that may confound the results are, for example, smoking tobacco, physical activity, and overweight and obesity. The intake of fruit and vegetables could also partly be a marker of a healthy diet in general characterised by, for example, high intakes of whole grains/cereal, legumes and nuts and low intakes of red and process meat, sugar-sweetened beverages and refined grains (75). Even though estimates used in meta-analyses are adjusted for factors like these, the extent of adjustments can vary between individual studies. Furthermore, in analyses based on thoroughly adjusted estimates, effects from residual confounding cannot be ruled out, considering also uncertainties involved in assessing dietary intake. Confounding factors are also an aspect considered when different grading systems are used for evaluating the evidence.

Observational studies on intermediate disease risk markers as well as trials on biomarkers for diseases may provide further support regarding causality. However, for vegetables, fruits, and berries as a food group, this type of evidence seems to be more limited. Observational studies suggest inverse associations between the intake of fruit and vegetables and the risk of overweight/obesity and hypertension, but the evidence was weak. Inverse associations are also seen regarding the metabolic syndrome; however, these are mainly based on cross-sectional studies. Trials, on the other hand, are commonly focused on specific fruits and vegetables, especially berries, and often in concentrated forms, such as juice, purée, or powder, rather than vegetables, fruits, and berries as a food group. They may also involve subjects with existing risk factors. Still, beneficial effects on inflammatory markers and cardiovascular risk factors, especially from berries, seen in trials support the assumption that vegetables, fruits, and berries contain substances that also could mediate protective effects on chronic diseases seen in observational studies. The role of dietary fibre and the numerous possible health effects of phytochemicals present in vegetables, fruits, and berries, as well as studies using biomarkers of these foods showing reduced risk of type 2 diabetes, cardiovascular disease, cancer, and all-cause mortality (see *Mechanisms*), give further support for a causal effect of fruit and vegetables intake.

Considering the current intakes of vegetables, fruits and berries in the Nordic and Baltic countries, which are below 400 g per day (16), and the potential health benefits of increasing these intakes, the current evidence strengthens the support for this recommendation. Recent meta-analyses have also indicated that different subgroups of vegetables, fruits, and berries such as green leafy vegetables, cruciferous vegetables, pommes fruit (apples and pears), and citrus fruits may have greater benefits than other subgroups. Although these analyses are based on few studies, it supports a recommendation to consume a variety of vegetables, fruits, and berries. Consuming a variety of these foods would also contribute with a wide range of nutrients and phytochemicals, since these contents differ between the different types of vegetables, fruits, and berries. Although the definitions of vegetables, fruits, and berries may differ, legumes are commonly not included, whereas potatoes and fruit juice may have been included. Excluding potatoes and other starchy vegetables and emphasising a variety of vegetables, fruits, and berries is also in line with the recommendation from WCRF/AICR, which is to eat at least five portions of servings per day (at least 400 g) of a variety of non-starchy vegetables and fruit; this includes non-starchy vegetables and fruits of different colours as well as non-starchy roots and tubers (76).

Most of the presented health outcomes generally appear later in life and few systematic reviews were found regarding the younger population. According to a qSR on dietary patterns (75), fruit and vegetables as components of a dietary pattern are associated with lower risk of cardiovascular disease and overweight and obesity in children, although the evidence was graded as limited. Considering the nutritional benefits of vegetables, fruits and berries and that dietary patterns seem to be established early in life (77, 78), these foods should be an important part of healthy diets also for children and adolescents.

Areas for future research include prospective studies on many of the outcomes and intermediate risk factors for which the evidence is still limited, as well as using biomarkers to complement dietary intake assessment. Metabolomic analysis is also an emerging field presenting new objective ways of assessing dietary exposures and diet-health associations (79). Possible health effects of different subgroups of fruit and vegetables need further investigation, which could lay ground for more specific recommendations in the future. The role of phytochemicals, including their bioavailability and interactions, is also an area for further research to better understand the mechanisms involved in the health effects of vegetables, fruits, and berries. Effects of different preparation methods, such as cooking and drying, may also be further investigated.

This scoping review has some limitations. The screening process was not done in duplicate and studies may have been missed, but selection and extractions were double-checked. The search strategy was focused on systematic reviews and meta-analyses only, and in areas where there are few such studies, original studies might have added further information. Studies using blood concentrations of antioxidants, which may be used as biomarkers for fruit and vegetable intake, were not included in the main search, although three relevant studies are referred to in the mechanisms section (72–74) of which one is a meta-analysis (72). The search strategy also focused on vegetables, fruits, and berries as a food group, and not on their subgroups or individual foods, which is in accordance with the scope and purpose of this review. However, a closer investigation of subgroups of fruit and vegetables could have shed further light on their diversity and specific characteristics. Overall, it seems unlikely that the main findings and general conclusions would be substantially affected by these limitations.

## Conflict of interest and funding

The authors have not received any funding or benefits from industry to conduct this study, and report no conflicts of interests. The authors received a small reimbursement from the Norwegian Directorate of Health for work linked to this article.
